# The filarial and the antibiotics: Single or combination therapy using antibiotics for filariasis

**DOI:** 10.3389/fcimb.2022.1044412

**Published:** 2022-11-17

**Authors:** Fatima Amponsah Fordjour, Alexander Kwarteng

**Affiliations:** ^1^ Department of Microbiology, University for Development Studies (UDS), Tamale, Ghana; ^2^ Department of Biochemistry and Biotechnology, Kwame Nkrumah University of Science and Technology (KNUST), Kumasi, Ghana; ^3^ Kumasi Centre for Collaborative Research in Tropical Medicine, Kwame Nkrumah University of Science and Technology (KNUST), Kumasi, Ghana

**Keywords:** filariasis, antibiotics, mass drug administration, neglected tropical diseases, *Wolbachia*

## Abstract

Filarial infections caused by nematodes are one of the major neglected tropical diseases with public health concern. Although there is significant decrease in microfilariae (mf) prevalence following mass drug administration (IVM/DEC/ALB administration), this is transient, in that there is reported microfilaria repopulation 6-12 months after treatment. *Wolbachia* bacteria have been recommended as a novel target presenting antibiotic-based treatment for filarial disease. Potency of antibiotics against filarial diseases is undoubtful, however, the duration for treatment remains a hurdle yet to be overcome in filarial disease treatment.

## Introduction

Filarial infections caused by nematodes mainly include: lymphatic filariasis (LF) and onchocerciasis (Oncho), causing severe morbidities such as elephantiasis, skin diseases and blindness (Litt et al., 2012). Globally, LF is endemic in 73 countries in Africa, Asia, and the Americas. Approximately 1.1 billion people are exposed to the infection, and more than one third of these individuals are in sub-Saharan Africa (Who, 2013). About 120 million individuals are affected globally with approximately 40 million individuals disabled owing to filariasis-related morbidity (Who, 2013).

Blood nematodes cause LF infection in the host’s lymphatic vessel. There are three species of these filarial worms: *Wuchereria bancrofti*, which accounts for almost 90% of the cases, with the remaining 10% caused by *Brugia malayi* and *Brugia timori*. Adult filarial parasites are located in the lymph vessels and are sexually dimorphic, producing thousands of first-stage larvae (microfilaria) for up to 8 years (Pfarr et al., 2009). Mosquito vectors of the genera *Aedes*, *Anopheles*, *Culex* or *Mansonia* are required for development of the larvae into the human infective stage, and for transmission to the human hosts.

The mainstay preventive intervention for these infections is based on mass drug administration (MDA) using diethylcarbamazine (DEC), ivermectin (IVM) and albendazole (ALB) (Simonsen et al., 2010). Although there is significant decrease in microfilariae (mf) prevalence following MDA, this is transient, in that there is repopulation of host system with microfilariae 6-12 months after MDA (Klarmann–schulz et al., 2017). What accounts for this observation is currently unclear but could be due to the fact that the MDA drugs do not target the adult worms rather the drugs disrupt embryogenesis, depriving the adult worm of mf production for some time (Volkmann et al., 2003; Klarmann–schulz et al., 2017). For this reason, MDA is given annually or biannually with the aim of terminating transmission. Although targeting mf through MDA has been successful in eliminating LF and Oncho in some countries (Egypt, Malawi, Vietnam, Yemen, etc), the program has not been feasible in other countries (Gabon, Equitorial, Guinea, South Sudan, etc), especially in developing countries co-endemic with Oncho/*Loa loa* (Traore et al., 2012; Katabarwa et al., 2013; Sodahlon et al., 2013; Wanji et al., 2015). The use of IVM in these countries can result in severe adverse reactions (SAEs) caused by drug induced death and associated inflammation of blood-borne *L. loa* mf in the brain (Boussinesq et al., 2006). Recently, a clinical trial administering a single triple-dose combination of IVM/DEC/ABZ has proven to have superior microfilaricidal effect in bancroftian filariasis for almost a year (Thomsen et al., 2016). These promising results have the potential to accelerate LF elimination goals, however, the drug combinations pose severe adverse effects in situations where LF co-exist with *Loa loa*/Oncho (Gardon et al., 1997; Thomsen et al., 2016).

Furthermore, epidemiological simulations have also predicted that MDA will not interrupt transmission in certain scenarios (Turner et al., 2010; Stolk et al., 2015). Alternative chemotherapy with a short course of treatment delivering safe macrofilaricidal effect is therefore imperative to achieve the Global Program to Eliminate Lymphatic Filariasis (GPELF) target (Taylor et al., 2010).

### Anti-*Wolbachia* approach

*Wolbachia* is an endosymbiotic bacteria (order Rickettsiales) present in most arthropods. Most insect species (20-80%) are estimated to be infected with these Gram-negative alpha-proteobacteria (Hilgenboecker et al., 2008). *Wolbachia* has been recommended as a possible target for antibiotic-based treatment or a novel anti-symbiotic chemotherapy (Hoerauf et al., 2000; McGarry et al., 2004). The association between *Wolbachia* and the filarial worm has been extensively explored, thus suggesting a symbiotic relationship between the two (Mclaren et al., 1975; Hoerauf et al., 2000; McGarry et al., 2004). This association has been therefore recommended as a novel target presenting antibiotic-based treatment for filarial disease. Tetracyclines (doxycycline and minocycline) are known to be efficacious against Rickettsiae bacteria by disrupting/interfering with protein synthesis pathway in the bacteria (Hoerauf et al., 2000). Albeit there is no uncertainty in potency with the class tetracyclines, the duration for treatment remains a hurdle yet to overcome in filarial disease treatment.

## Antibiotics used in filarial clinical trials

For over a decade *Wolbachia* has become a target in the treatment of filarial disease. This has led to several field trials evaluating the efficacy of the various classes of antibiotics including: tetracyclines (minocycline, doxycycline), rifamycin (rifampicin, rifapentine), azithromycin, chloramphenicol, etc.

In LF and Oncho field trials, tetracyclines have been shown to have biological effect on *Wolbachia*, thus, the depletion of the bacteria in the worm obstructs early embryogenesis and female worm development (Hoerauf et al., 2000).

### Doxycycline (DOX)

Doxycycline is well-tolerated, safe, inexpensive, and is commonly used as an antibiotic and anti-inflammatory drug for the treatment of a variety of medical conditions. Doxycycline displays excellent activity against Gram-positive and Gram-negative aerobic and anaerobic pathogens. The antibiotic activity of doxycycline mainly targets growth of bacteria by conformationally binding to the 30S prokaryotic ribosomal unit during protein synthesis resulting in the blockage of aminoacyl-tRNA binding to the mRNA-ribosome complex.

Doxycycline has been found to be anti-inflammatory in nature in filarial diseases (Awata et al., 2002; Debrah et al., 2006; Mand et al., 2012), thus reducing excessive inflammation that could be detrimental to the health of individuals. The anti-inflammatory property of doxycycline is due to its slow anti-filarial mode of action (killing worms slowly), and hence can be used in *L*. *loa* endemic areas, since this filarial parasite does not contain *Wolbachia* (Turner et al., 2006; Turner et. al., 2010). A six-week course of doxycycline treatment among individuals resulted in tremendous reduction in microfilariae, and a significant amelioration in patient’s condition (Mand et al., 2012; Coulibaly et al., 2012; Debrah et al., 2019). Further studies with doxycycline treatment has been shown to be effective in mediating long-term reductions of microfilaremia in a population infected with *Mansonella perstans*, another form of filarial disease (Awata et al., 2002; Mand et al., 2012). Confirmatory study on doxycycline treatment has shown that there is sustained suppression of microfilariae 36 months after treatment, suggesting that doxycycline has a sustained effect on adult filarial worm (Coulibaly et al., 2012). Despite the success seen in doxycycline treatment, there is still the need of finding drug(s) that can be delivered on a short course, as the six/four weeks course for doxycycline treatment is usually not feasible with national programs (logistical challenge), and also does not encourage compliance (Debrah et al., 2009; Debrah et al., 2019). Moreover, the contraindications of doxycycline seen in pregnant women and children present challenges to widespread scale-up programs in resource-poor settings of Sub-Saharan Africa (Czeizel and Rockenbauer, 1997). Howbeit, in areas of partial elimination of filarial disease, doxycycline can be used in test and treat scenarios to achieve end game target for the disease.

### Minocycline (MIN)

Minocycline is a semi-synthetic, second-generation drug belonging to the tetracycline family. It is effective against Gram-positive, Gram-negative, and many atypical bacteria including *Mycobacterium* species and *Bacillus anthracis* (Upadhya et al., 2018). Minocycline has the similar mechanism of action as DOX. Through pharmacokinetic–pharmacodynamic (PK-PD) modelling, studies have demonstrated that the related second-generation tetracycline (minocycline) may reduce overall treatment time compared with doxycycline against the human filarial parasite *Brugia malayi* in a severe combined immunodeficiency (SCID) mouse model (Czeizel and Rockenbauer, 1997; Debrah et al., 2011). This data further suggest that not only can minocycline be used as alternative to doxycycline in test and treat strategies, it may offer some advantages through either improved *Wolbachia* kill rates or lower dosage requirements, which may impact on tolerability or safety profiles (Sharma et al., 2016). Further, human clinical trial administering minocycline for 3 weeks reported the drug to be more efficacious than doxycycline administered over the same course (Klarmann–schulz et al., 2017). Although the incidence of adverse effects (AEs) associated with either drug (doxycycline or minocycline) is very low, there is a slightly higher incidence of AE with minocycline (Smith and Leyden, 2005). The shorter treatment period may offset this slight increase in risk of AEs with minocycline. It will now be important to undertake clinical evaluations of minocycline, supported through full PK/PD analysis, to confirm these benefits. Although minocycline has been identified as a superior anti-*Wolbachia* agent to doxycycline in the *B. malayi* SCID mouse model (Sharma et al., 2016), the same contraindications seen in doxycycline apply because they are in the same class of antibiotics.

### Rifampicin (RIF)

Rifampicin is active against Gram-positive cocci but not on enterobacteriaceae (such as *E. coli, Klebsiella, Salmonella or Pseudomonas*) (Jammal et al., 2015). Rifampicin is thought to inhibit bacterial DNA-dependent RNA polymerase, which appears to occur as a result of drug binding in the polymerase subunit within the DNA/RNA channel, facilitating direct blocking of the elongating RNA.

In an attempt to focus on finding shorter treatment course or other antibiotics that are practical for use in MDA, RIF has been explored in *in vitro/in vivo* LF and Oncho mice studies to exhibit superior anti- *Wolbachia* potency compared with the tetracyclines (Smith and Leyden, 2005; Halliday et al., 2014; Klarmann–schulz et al., 2017; Debrah et al., 2019). However, this success has not yet been translated into human clinical trials. A minimum RIF dose calculation of 30–40 mg/kg in humans is required to deplete *Wolbachia* beyond the 90% threshold predictive of clinical cure (Smith and Leyden, 2005; Debrah et al., 2009). To buttress this evidence, fourfold dose elevations of RIF have recently been identified as safe when delivered for periods of 1 month (Boeree et al., 2017) in patients with TB, indicating that RIF at a high dose could be deployed as a short-course for macrofilaricidal effect in humans (Katabarwa et al., 2013). This drug therefore has the potential to achieve *Wolbachia* depletions predictive of cure when administered for 1-2 weeks against filarial parasites (Aljayyoussi et al., 2017). Combinatorial effect of filarial antibiotics has shown that 2-week doxycycline plus rifampicin could have stronger macrofilaricidal activity (Debrah et al., 2011) compared to single dose doxycycline.

### Azithromycin

Azithromycin binds to the 23S rRNA of the bacterial 50S ribosomal subunit. It stops bacterial protein synthesis by inhibiting the transpeptidation/translocation step of protein synthesis and by inhibiting the assembly of the 50S ribosomal subunit. Although macrolides are known to have better efficacy against *Wolbachia* (Townson et al., 2000; Debrah et al., 2009), azithromycin like the DNA-gyrase inhibitors (quinolone) has failed in several field trials in giving significant effect against *Wolbachia* (Hoerauf et al., 2008; Hansen et al., 2011; Aliaa et al., 2019). Initially, *in vitro* study assessing the effect of azithromycin on *B. malayi* resulted in a partial inhibition of molting of the parasite (Rao, 2002). Other study has shown that a six-week trial of azithromycin monotherapy in humans proved futile as there was no significant reduction of worm load after 12 months of treatment at either 250 mg/day or 1.2 g/per week (Hoerauf et al., 2008). Another study using rifampicin/azithromycin alone or both, neither showed *Wolbachia* depletion nor anti-filarial effect after a short course of 5 days. These studies confirmed that azithromycin is less effective in treating human filariasis.

### Moxifloxacin and rifapentine

Rifapentine is an antibiotic drug used in the treatment of tuberculosis. It inhibits DNA-dependent RNA polymerase activity in susceptible cells. Specifically, it interacts with bacterial RNA polymerase but does not inhibit the mammalian enzyme (Micromedex, 2022). Moxifloxacin is a novel fourth-generation fluoroquinolone with a broad spectrum of antibacterial activity against Gram-positive and Gram-negative bacteria, anaerobes, and atypical organisms (Scholar, 2007). Moxifloxacin is bactericidal and its mode of action depends on blocking of bacterial DNA replication by binding itself to an enzyme called DNA gyrase, which allows the untwisting required to replicate one DNA double helix into two. Moxifloxacin and rifapentine have progressed into human filarial clinical trials. Both are currently been experimented in phase 2 randomized open-label clinical trials at a dosage of 900 mg/day rifapentine plus 400 mg/day moxifloxacin for 14 or 7 days [Debrah and Klarmann-Schulz U (2018)]. Previously, *in vitro* and *in vivo* experiments using moxifloxacin and rifapentine showed that this combination administered for only 4-7 days significantly could deplete *Wolbachia* load in adult worms (Specht et al., 2018).

### Corallopyronin

Corallopyronin, originally isolated from the myxobacterial strain *Corallococcus coralloides*, is a natural compound and a non-competitive RNA Polymerase (RNAP) inhibitor (Irschik et al., 1985; Schäberle et al, 2014; Schiefer et al., 2012). It has many bioactivities including microfilaricidal. The genome of *Wolbachia* found in filarial nematodes encode complete RNAP, which is a suitable target for antibiotics. *In vitro* and *in vivo* studies in animal models have shown the potential of corallopyronin against *Wolbachia* with a good safety profile (Debrah et al., 2014; Specht et al., 2018). However, further clinical trials in humans will be required to examine the efficacy of corallopyronin as a treatment regimen in patients with onchocerciasis and LF.

### Berberine, rapamycin and globomycin

Berberine (Li et al., 2011), rapamycin (Voronin et al., 2012) and globomycin (Johnston et al., 2010) are currently in the initial phase of experimental research in filarial trials (Aliaa et al., 2019).

Besides antibiotics, several non-antimicrobial compounds such as anti-oxidants, anti-histamines and synthetic compounds have demonstrated activity against *Wolbachia in vitro*, opening up the potential for other anti- *Wolbachia* therapeutic combinations to be explored and exploited in future research (Johnston et al., 2014).

An example of such anti- *Wolbachi*a candidate is AWZ1066S (Hong et al., 2019). AWZ1066S has superior efficacy compared to existing anti-*Wolbachia* therapies in validated preclinical models of infection and has drug metabolism and pharmacokinetics (DMPK) characteristics that are compatible with a short therapeutic regimen of 7 days or less. The main setback with the current MDA is macrofilaricidal effect and regimen duration. This candidate molecule, however, is well-positioned for onward development and has the potential to make a significant impact on communities affected by filariasis, delivering safe macrofilaricidal effect within a short treatment course. AWZ1066S has been demonstrated to be capable of depleting *Wolbachia >*90% with sustained sterilization of microfilaria production in two independent filarial infection models over a week treatment course.

In an attempt to find superiority for AWZ1066S over other anti-*Wolbachia* candidates such as moxifloxacin, rifampicin, minocycline and doxycycline, a time-kill assay was structured from standard microfilaria assay in a panel of anti-*Wolbachia* drugs (McGarry et al., 2004; Voronin et al., 2012), screened over a varied exposure time intervals for 1, 2 or 6 days (Hong et al., 2019). Findings from this assay suggested that AWZ1066S can achieve maximum reduction of *Wolbachia* just after 1 day of drug exposure compared with the antibiotics tested.

Moreover, AWZ1066S meets all target candidate profile criteria for an anti-*Wolbachia* macrofilaricidal drug and has entered formal preclinical evaluation. This compound may provide a unique opportunity to make a vital contribution to communities affected by filariasis, importantly after confirmation of the macrofilaricidal effect of AWZ1066S in human clinical trials.

## Possible pathways targeted by antibiotics used in filarial trials

The action of antibiotics has shown to target specific pathways that deplete *Wolbachia* bacteria in filarial infection (Landmann et al., 2011). An example is the apoptosis of adult germline cells, embryos and somatic cells of microfilariae in animals/*in vitro* studies following depletion of *Wolbachia* of *B. malayi* after tetracycline treatment (Landmann et al., 2011).

Furthermore, apoptosis has also been observed in the uterine tissues of *O. volvulus* 21 months following antibiotic treatment of people with onchocerciasis. To buttress the importance of antibiotic in depleting the symbiotic bacteria (*Wolbachia)*, a study investigated treatment of antibiotic on the *Wolbachia*-free filarial nematode, *Acanthocheilonema viteae* and found out that it has no effect on the viability or biological processes of the species (Hoerauf et al., 1999). This further confirms findings that apoptosis is due to the loss of *Wolbachia* rather than a direct effect of tetracycline treatment (Landmann et al., 2011).

Studies have shown that the first event which occurs after *Wolbachia* depletion from adult female worm is the blockage of embryogenesis and cessation of microfilariae production, which is consistent with the extensive and profound apoptosis observed in uterine embryonic stages of *B. malayi* and onchocerciasis (Taylor et al., 2000; Misra et al., 2007). This results in long-term sterilization of adult female worms and the slow decline in microfilariae levels, until patients reach a sustained amicrofilariae state, with benefits to disease reduction and interruption of transmission in both onchocerciasis and lymphatic filariasis.

Another positive target of the *Wolbachia* bacteria in filarial nematodes is their RNA polymerase (RNAP). Bacterial RNAP has been proven to be a potent target for the development of highly specific antibacterial drugs as it is a vital enzyme for cell survival (Brodolin, 2018). RNAP and its associated transcription factors are highly conserved across the bacterial domain and represent excellent targets for broad spectrum antibacterial agent discovery. Classes of antibiotics that inhibit RNAP include: rifamycins, sorangicin, streptolydigin and myxopyronin. Rifamycin antibiotics (rifapentine and rifampicin) are widely used to treat a variety of infections including tuberculosis. Rifampicin and rifapentine have already been proven to be efficacious against *Wolbachia* bacteria in filarial mouse studies (Rao, 2002; Smith and Leyden, 2005; Debrah et al., 2019). Studies have indicated that rifampicin bind within the cleft close to the active centre of RNAP, which sterically hinders growth of the RNA product eventually inhibiting RNA synthesis sites (Ma et al., 2016).

## Time effectiveness of the various anti-filaria either singly or combinations

IVM is the standard drug for treatment of onchocerciasis while ALB and IVM/DEC are for LF in filarial endemic communities. Nonetheless, these drugs have been used in triple dose combinations (IVM/ALB/DEC) to achieve superior anti-filarial effect in LF (Thomsen et al., 2016; Winnen et al., 2002). This notwithstanding, the impressive response achieved with this combination cannot be administered in areas co-endemic with Oncho/*Loa loa*, as this poses severe threat. Through *in vitro* studies/*in vivo*, rifampicin has been found to be potent against filarial infection. More so, its combination with albendazole has been proven to be superior when given in filarial field trials (Smith and Leyden, 2005; Halliday et al., 2014; Debrah et al., 2019). Furthermore, doxycycline plus rifampicin has also been shown to have significant effect on filarial infection in mouse study compared to doxycycline alone (Volkmann et al., 2003). This study further explains that doxycycline plus rifampicin can be administered for 14 days, and still show macrofilaricidal effect, instead of doxycycline alone for 4 or 6 weeks which may affect compliance.

There have been several suggestions and discussion about drug combinations (antibiotic or anti-helminthic combinations) with regards to having superior effect compared to single doses (**Table 1**). All these antibiotics have been shown to deplete Wolbachia bacteria thereby resulting in the disruption of the embryogenesis of female filarial worms. However, IVM, ALB and DEC are anti-helminths and therefore does not target the *Wolbachia* bacteria but rather target microfilariae reduction (Katabarwa et al., 2015). Anti-helmintic drugs act rapidly and selectively on neuromuscular transmission of nematode by depleting the parasite of energy thereby leading to paralysis (Martin, 1997).

**Table 1:** Anti-filarial drugs and their time effect efficacies against the various forms of filarial diseases.

Drug	Dosage	Regimen Duration	Disease	Reference
Ivermectin (IVM)	150 ukg	annually	Onchocerciasis	(World Health Organization, 2004)
Albendazole (ALB)+IVM	ALB 400 mg+IVM 150ukg	annually	Lymphatic filariasis (LF)	(World Health Organization, 2004)
ALB + Doxycycline (DOX)	200 mg/d ALB 800 mg/d + DOX	ALB 3 days + DOX 3 weeks	Onchocerciasis	(Klarmann–schulz et al., 2017)
ALB	800 mg/d	3 days	Onchocerciasis	(Klarmann–schulz et al., 2017)
DOX	200 mg/d	4 weeks	Lymphatic filariasis	(Debrah et al., 2006; Klarmann–schulz et al., 2017)
DOX	200 mg/d	6 weeks	Lymphatic filariasis	(Debrah et al., 2006; Mand et al., 2012)
DOX	100 mg/d	6 weeks	Onchocerciasis	(Debrah et al., 2015)
DOX	200 mg/d	6 weeks	*Mansonella perstans*	(Debrah et al., 2019)
DOX + Rifampicin (RIF)	DOX 200 mg/d + RIF 10 mg/kg/d	2 weeks	Lymphatic filariasis	(Debrah et al., 2011)
RIF	20 mg/kg/d	5 days	Onchocerciasis	(Richards et al., 2007)
RIF	35 mg/kg/d	2 weeks	Preclinical LF and Onchocerciasis	(Aljayyoussi et al., 2017)
Minocycline (MIN)	200 mg/d	3 weeks	Onchocerciasis	(Klarmann–schulz et al., 2017)
MIN	25 mg/kg x2 daily	4 weeks	LF Murine	(Sharma et al., 2016)
Moxidectin (MOX)	8 mg	1 time	Onchocerciasis	(Opoku et al., 2018)
MOX	4 mg	1 time	Onchocerciasis	(Awadzi et al., 2014; Turner et al., 2015)
MOX	2 mg	1 time	Onchocerciasis	(Awadzi et al., 2014; Turner et al., 2015)
Azithromycin (AZT)	12 mg/kg/d	5 days	Onchocerciasis	(Richards et al., 2007)
AZT	250 mg/d	6 weeks	Onchocerciasis	(Hoerauf et al., 2008)
AZT	1.2 g	1 week	Onchocerciasis	(Hoerauf et al., 2008)
Tetracycline	20l g/ml	2 days	*Brugia malayi in vitro*	(Sabharwal and Anandharaman, 2010)
Moxifloxacin (MOXI)+ Rifapentine (RIFA)	RIFA 900 mg/d + MOXI 400 mg/d	1 week/2 week	*in vitro* and *in vivo*	(Specht et al., 2018)

## Practical application of antimicrobial drugs for filariasis in Africa: Antimicrobial resistance a common challenge in the population

Vector-borne diseases remain an important challenge for public health globally, and contribute to over 17% of the globally estimated burden of all infectious diseases (WHO, 2017b). Reports have shown an encouraging progress in the control of some important mosquito borne diseases including malaria (Cibulskis et al., 2016) and filariasis (Cromwell et al., 2020). This notwithstanding, there remain certain challenges regarding the controlling of these diseases, with the evolution of resistance against anti-parasitic drugs being one of the most important (Conrad and Rosenthal, 2019).

Anti-filarial resistance has become alarming in larger populations since the inception of GPEL, probably leading to the WHO’s inability to achieve target (McCarthy, 2005). Ivermectin resistance has been reported in Ghana, with the widespread of benzimidazole resistance (such as albendazole) present due to specific mutations in the gene encoding β-tubulin associated with drug resistance (Anderson and Jaenike, 1997). Moreover, it has been shown that DEC susceptibility is not 100% for lymphatic filariasis treatment. A review of the mechanisms of resistance to these anti-helminthics is therefore imperative to optimize the treatment for human lymphatic filariasis.

There has been data on the antimicrobial resistance (AMR) in Africa with high level of resistance to the commonly used antibiotics in the Sub-Saharan African region (Leopold et al., 2014). For example, 90% of Gram negatives have been shown to be resistant to chloramphenicol, a commonly used antibiotic. In contrast, resistance to third-generation cephalosporins (like ceftriaxone) was less common, recommending this group for use (Leopold et al., 2014). To design suitable local and global interventions, it is important to understand the status of AMR and identify knowledge gaps.

The discovery of drug resistance in veterinary parasitology indicates that the resistance to the treatment of lymphatic filariasis in human nematode parasites could also exist. Although it is urgent to prove anti-helminthic resistance in filarial parasites (*W. bancrofti, Brugia spp, O. volvulus)*, it is difficult to investigate this because these parasites do not have free-living stages and therefore cannot be cultured in animal models (Cobo, 2016). This clearly shows that parasitological evidence of anti-helminthic drug resistance can only be available by demonstrating the persistence of parasites after treatment over a long period of time.

Studies have shown that antimicrobials given in combinations have higher efficacies than those given singly (Schwab et al., 2005). Treatment with anti-filarial drugs through the MDA programme has proved to be significant in the reduction of infection and morbidity, although in some parts of Asia, the program over 50 years could not eradicate the disease (Pichon, 2002). Nonetheless, the continual use of the three anti-helminthics used for filarial treatment increases the risk for the emergence and spread of drug resistance, which may be a threat to the set goals of the GPELF. Since there is no available vaccine, and the vector control programs have failed, the short-term goal is to identify and develop new classes of drugs and alternative chemotherapeutic strategies. The *Wolbachia* bacteria has been identified in filaria parasites over some decades now (Andre et al., 2002). Many antibiotics but, above all doxycycline are known to be effective in the treatment of human filariasis through the depletion of *Wolbachia* (mclaren et al., 1975; Gardon et al., 1997; Hoerauf et al., 2000). Despite the efficacy of doxycycline against filarial disease, there is contraindication of the drug in pregnant/breastfeeding women and children under 9 years, therefore there is the need for new drugs that will be safe for these populations (Jammal et al., 2015; Jimah T, 2020).

## The way forward in filarial disease elimination

A meeting held by WHO in Geneva 1999, primarily was about finding suitable initiative to identify new molecular targets for anti-filarial chemotherapy. One of the drug targets discovered was the endosymbiotic bacteria *Wolbachia*. Although it has been over two decades since the *Wolbachia* bacteria was discovered in filarial parasites (mclaren et al., 1975), its significance as a potential target unravelled a little later.

Initially the GPELF’s goal was in two folds: the first was interrupting transmission through mass drug administration (IVM, DEC and ALB). The second goal was to provide morbidity management for individuals with pathologies of the disease. It was the GPELF’s target to eliminate LF by 2020. However, the disease is still of public health concern. The disease is endemic in 73 countries with 1.39 billion people at risk of infection globally (WHO, 2011). Out of 73 countries endemic for LF, 20 have stopped interventions after passing the first transmission assessment survey (TAS) and are conducting surveillance to verify elimination. Additional 30 countries have delivered MDA at least once in all endemic areas and are also on track to achieve elimination (WHO, 2017a). Factors that might impede the success of the program may be low coverage that may be sustained over multiple treatment rounds and low compliance primarily due to adverse effects of the drug. The required duration of MDA has turned out to be longer than the anticipated years, which might be due to relatively high baseline mf prevalence levels.

Antibiotics have gained popularity in the treatment of filarial diseases over the years (Debrah et al., 2006; Debrah et al., 2007; Debrah et al., 2011; Mand et al., 2012; Sharma et al., 2016; Klarmann–schulz et al., 2017). Compared to the standard MDA drugs (IVM, DEC and ALB), antibiotics used in treating filarial diseases take longer, usually 2-6 weeks. Reports from field trials with tetracyclines suggested at least 3 weeks to achieve macrofilaricidal effect (mclaren et al., 1975). Despite their potency in reducing microfilariae levels and depleting the *Wolbachia* bacteria, their longer regimen duration makes them not feasible with national scale-up programs. Although the treatment duration for rifampicin (1-2 weeks) might be shorter compared to the tetracyclines, this is still long and might pose logistic challenge and affect compliance. There is therefore the need for further studies in finding drug(s) of choice, that has comparatively shorter treatment duration and has macrofilaricidal effect.

## Conclusion

Looking at the successes achieved from the various programs for eliminating filarial disease (onchocerciasis control program (OCP) and global program to eliminate lymphatic filariasis (GPELF), there has been tremendous improvement in some countries. Other countries especially those in the Sub-Saharan African region have not yet achieved target. It is therefore imperative to:

1. Explore new anti-filarial drug(s) with safe safety profile, shorter treatment time, as well as macrofilaricidal effect.
2. Further research should be directed towards other classes of antibiotics to ascertain their potency and regimen duration in filarial infections.
3. Other non-antimicrobials should be investigated to help achieve WHO’s target of elimination.

## Author contributions

FAF: Conceptualization, literature review, title and abstract review, full-text review, data extraction, manuscript writing, revision, and submission. AK: Conceptualization, manuscript writing, supervision, proof-reading, and revisions. All authors contributed to the article and approved the submitted version.

## Acknowledgments

We would like to acknowledge the authors of the referenced studies.

## Conflict of interest

The authors declare that the research was conducted in the absence of any commercial or financial relationships that could be construed as a potential conflict of interest.

## Publisher’s note

All claims expressed in this article are solely those of the authors and do not necessarily represent those of their affiliated organizations, or those of the publisher, the editors and the reviewers. Any product that may be evaluated in this article, or claim that may be made by its manufacturer, is not guaranteed or endorsed by the publisher.

## Abbreviations

MDA, mass drug administration; NTDs, neglected tropical diseases; WHO, world health organization; TAS, transmission assessment survey; OCP, onchocerciasis control program, GPELF, global programme to eliminate lymphatic filariasis; IVM, Ivermectin; ALB, Albendazole; DEC, Diethylcarbamazine.

## References

[B1] Aliaa W. Sulaiman W. Kamtchum–tatuene J. Mohamed M. H. Ramachandran V. Ching S. M. . (2019). Anti– wolbachia therapy for onchocerciasis & lymphatic filariasis: Current perspectives. Indian J Med Res 149(6), 706–714. doi: 10.4103/ijmr.IJMR_454_17 31496523 PMC6755775

[B2] Aljayyoussi G. Tyrer H. E. Ford L. Sjoberg H. Pionnier N. Waterhouse D. . (2017). Rifampicin achieves wolbachia depletion predictive of curative outcomes in preclinical models of lymphatic filariasis and onchocerciasis. Sci. Rep. 7 (1), 1–11. doi: 10.1038/s41598-017-00322-5 28303006 PMC5428297

[B3] Anderson T. J. Jaenike J. (1997). Host specificity, evolutionary relationships and macrogeographic differentiation among ascaris populations from humans and pigs. Parasitology. 115, 325–342. doi: 10.1017/S0031182097001339 9300471

[B4] Andre A. S. Blackwell N. M. Hall L. R. Hoerauf A. Bratting N. W. Volkmann L. . (2002). The role of endosymbiotic wolbachia bacteria in the pathogenesis of river blindness. Science 295 (5561), 1892–1895. doi: 10.1126/science.1068732 11884755

[B5] Awadzi K. Opoku N. Attah S. Al E. (2014). A randomized, single–ascending–dose, ivermectin–controlled, double–blind study of moxidectin in onchocerca volvulus infection. PloS Negl. Trop. Dis. 8 (6), e2953. doi: 10.1371/journal.pntd.0002953 24968000 PMC4072596

[B6] Awata T. Inoue K. Kurihara S. Ohkubo T. Watanabe M. Inukai K. . (2002). A common polymorphism in the 5’–untranslated region of the VEGF gene is associated with diabetic retinopathy in type 2 diabetes. Diabetes 51 (0012–1797), 1635–1639. doi: 10.2337/diabetes.51.5.1635 11978667

[B7] Boeree M. J. Heinrich N. Aarnoutse R. Diacon A. H. Dawson R. Rehal S. . (2017). High–dose rifampicin, moxifloxacin, and SQ109 for treating tuberculosis: A multi–arm, multi–stage randomised controlled trial. Lancet Infect. Dis. 17 (1), 39–49. doi: 10.1016/S1473-3099(16)30274-2 28100438 PMC5159618

[B8] Boussinesq M. Kamgno J. Pion SD G. J. (2006). What are the mechanisms associated with post–ivermectin serious adverse events? trends. Parasitol. 22, 244–246. 10.1016/j.pt.2006.04.006 16632406

[B9] Brodolin K. (2018). Antibiotics targeting bacterial RNA polymerase. Antibiot Targets, Mech Resist 12. doi: 10.1002/9783527659685.ch12

[B10] Cibulskis R. E. Alonso P. Aponte J. Aregawi M. Barrette A. Bergeron L. . (2016). Malaria: Global progress 2000–2015 and future challenges. Infect. Dis. Poverty 5, 1–8. doi: 10.1186/s40249-016-0151-8 27282148 PMC4901420

[B11] Cobo F. (2016). Review determinants of parasite drug resistance in human lymphatic filariasis. Revista Española de Quimioterapia 29 (6), 288–295. 27858056

[B12] Conrad M. Rosenthal P. (2019). Antimalarial drug resistance in Africa: The calm before the storm? Lancet Infect. Dis. 19, E338–E351. doi: 10.1016/S1473-3099(19)30261-0 31375467

[B13] Coulibaly Y. I. Dembele B. Diallo A. A. Ettie M. Lipner S. S. D. Doumbia S. S. . (2012). A randomized trial of doxycycline for mansonella perstans infection yaya. NIH Public access 361 (15), 1448–1458. doi: 10.1056/NEJMoa0900863.A PMC3410935 19812401

[B14] Cromwell E. A. Schmidt C. A. Kwong K. T. Pigott D. M. Mupfasoni D. Biswas G. . (2020). The global distribution of lymphatic filariasis, 2000–2018: A geospatial analysis. Lancet Glob Heal. 8, e1186–e1194. doi: 10.1016/S2214-109X(20)30286-2 PMC7443698 32827480

[B15] Czeizel A. E. Rockenbauer M. (1997). Teratogenic study of doxycycline. Obstet Gynecol 89, 524–528. doi: 10.1016/S0029-7844(97)00005-7 9083306

[B16] Debrah U. K. Klarmann-Schulz U . (2018). The efficacy of rifapentine plus moxifloxacin against onchocerciasis: A randomized, open label pilot trial. Available at: www.isrctn.com/ISRCTN43697583.

[B17] Debrah A. Y. Mand S. Marfo–debrekyei Y. Batsa L. Albers A. Specht S. . (2011). Macrofilaricidal activity in wuchereria bancrofti after 2 weeks treatment with a combination of rifampicin plus doxycycline. Journal of Parasitology Research 2011, 201617. doi: 10.1155/2011/201617 21687646 PMC3112504

[B18] Debrah A. Y. Mand S. Marfo–Debrekyei Y. Batsa L. Pfarr K. Lawson B. . (2009). Reduction in levels of plasma vascular endothelial growth factor–a and improvement in hydrocele patients by targeting endosymbiotic wolbachia sp. in wuchereria bancrofti with doxycycline. Am. J. Trop. Med. Hyg. 80 (6), 956–963. doi: 10.4269/ajtmh.2009.80.956 19478258

[B19] Debrah A. Y. Mand S. Specht S. Marfo–Debrekyei Y. Batsa L. Pfarr K. . (2006). Doxycycline reduces plasma VEGF–C/sVEGFR–3 and improves pathology in lymphatic filariasis. PloS Pathog. 2 (9), 0829–0843. doi: 10.1371/journal.ppat.0020092 PMC1564427 17044733

[B20] Debrah A. Y. Mand S. Toliat M. R. Marfo–Debrekyei Y. Batsa L. Nürnberg P. . (2007). Plasma vascular endothelial growth factor–a (VEGF–a) and VEGF–a gene polymorphism are associated with hydrocele development in lymphatic filariasis. Am. J. Trop. Med. Hyg. 77 (4), 601–608. doi: 10.4269/ajtmh.2007.77.601 17978056

[B21] Debrah L. B. Phillips R. O. Pfarr K. Klarmann–schulz U. Opoku V. S. Nausch N. . (2019). The efficacy of doxycycline treatment on mansonella perstans Infection: An open–label , randomized trial in Ghana. The American Journal of Tropical Medicine and Hygiene 101 (1), 84–92. doi: 10.4269/ajtmh.18-0491 31162017 PMC6609185

[B22] Debrah A. Y. Specht S. Klarmann–schulz U. Batsa L. Mand S. Marfo–debrekyei Y. . (2015). Doxycycline leads to sterility and enhanced killing of female onchocerca volvulus worms in an area with persistent micro fi laridermia after repeated ivermectin Treatment: A randomized ,Trial, double–blind. Clinical Infectious Diseases 61(4), 517–526. 25948064 10.1093/cid/civ363 PMC4518165

[B23] Gardon J. Gardon–wendel N. Kamgno J. Chippaux J. (1997). Serious reactions after mass treatment of onchocerciasis with ivermectin in an area endemic for Loa loa infection. 350. 10.1016/S0140-6736(96)11094-1 9217715

[B24] Halliday A. Guimaraes A. F. Tyrer H. E. Metuge H. M. Steven A. Wanji S. . (2014). A murine macrofilaricide pre–clinical screening model for onchocerciasis and lymphatic filariasis. Parasit Vectors 7, 472. doi: 10.1186/s13071-014-0472-z 25338621 PMC4212127

[B25] Hansen R. D. E. Trees A. J. Bah G. S. Hetzel U. Martin C. Bain O. . (2011). A worm ‘ s best friend : recruitment of neutrophils by wolbachia confounds eosinophil degranulation against the filarial nematode onchocerca ochengi 2293–2302. doi: 10.1098/rspb.2010.2367 PMC3119012 21177682

[B26] Hilgenboecker K. Hammerstein P. Schlattmann P. Telschow A. Werren J. H. (2008). How many species are infected with wolbachia? – a statistical analysis of current data. FEMS Microbiol. Lett. 281 (2), 215–220. doi: 10.1111/j.1574-6968.2008.01110.x 18312577 PMC2327208

[B27] Hoerauf A. Marfo–Debrekyei Y. Büttner M. Debrah A. Y. Konadu P. Mand S. . (2008). Effects of 6–week azithromycin treatment on the wolbachia endobacteria of onchocerca volvulus. Parasitol. Res. 103, 279–286. doi: 10.1007/s00436-008-0964-x 18421478

[B28] Hoerauf A. Nissen–pähle K. Schmetz C. Henkle–dührsen K. Blaxter M. L. Büttner D. W. . (1999). Tetracycline therapy targets intracellular bacteria in the filarial nematode litomosoides sigmodontis and results in filarial infertility. J. Clin. Investigation 103 (1), 1, 11–17. doi: 10.1172/JCI4768 PMC407866 9884329

[B29] Hoerauf A. Volkmann L. Hamelmann C. Adjei O. Auten–Rieth I. B. Fleischer B. . (2000). Endosymbiotic bacteria in worms as targets for a novel chemotherapy in filariasis. Lancet 355, 1242–1243. doi: 10.1016/S0140-6736(00)02095-X 10770311

[B30] Hong W. D. Benayoud F. Nixon G. L. Ford L. Johnston K. L. Clare R. H. (2019). AWZ1066S, a highly specific anti-Wolbachia drug candidate for a short-course treatment of filariasis. Proceedings of the National Academy of Sciences 116 (4), 1414–1419. doi: 10.1073/pnas.1816585116 PMC6347715 30617067

[B31] Irschik H. Jansen R. Höfle G. Gerth K R. H. (1985). The corallopyronins, new inhibitors of bacterial RNA synthesis from myxobacteria. J. Antibiot 38, 145–152. doi: 10.7164/antibiotics.38.145 2581926

[B32] Jammal J. Zaknoon F. Kaneti G. Goldberg K. Mor A. (2015). Sensitization of gram–negative bacteria to rifampin and OAK combinations. Sci. Rep. 5, 1–6. doi: 10.1038/srep09216 PMC4363860 25782773

[B33] Jimah T O. O. (2020). Socio–demographic characteristics of the association between knowledge of antibiotic therapy and prudent use in Ghana. J. Glob Heal Rep. 4, e2020034. doi: 10.29392/001c.12838

[B34] Johnston K. L. Ford L. Umareddy I. Townson S S. S. Pfarr K. Hoerauf A. . (2014). Repurposing of approved drugs from the human pharmacopoeia to target wolbachia endosymbionts of onchocerciasis and lymphatic filariasis. Int. J. Parasitol. Drugs Drug Resist. 4, 278–286. doi: 10.1016/j.ijpddr.2014.09.001 25516838 PMC4266796

[B35] Johnston K. L. Wu B. Guimarães A. Ford L. S. B. Taylor M. J. . (2010). Lipoprotein biosynthesis as a target for anti–wolbachia treatment of filarial nematodes. Parasit Vectors 3, 99. doi: 10.1186/1756-3305-3-99 20946650 PMC2964653

[B36] Katabarwa M. N. Habomugisha P. Eyamba A. Byamukama E. Nwane P. Arinaitwe A. . (2016). Community–directed interventions are practical and effective in low–resource communities: Experience of ivermectin treatment for onchocerciasis control in Cameroon and Uganda, 2004–2010. Int. Health 8 (2), 116–123. doi: 10.1093/inthealth/ihv038 26152231

[B37] Katabarwa M. Hassan H. K. Unnasch T. R. Mackenzie C. D. Richards F. Hashim K. (2013). Interruption of onchocerca volvulus transmission in the Abu hamed focus , interruption of onchocerca volvulus transmission in the Abu hamed focus, Sudan. Am. J. Trop. Med. Hygiene 89 (1), 51–57 doi: 10.4269/ajtmh.13-0112 PMC3748488 23690554

[B38] Klarmann–schulz U. Specht S. Debrah A. Y. Batsa L. Ayisi–boateng N. K. Osei–mensah J. . (2017). Comparison of doxycycline, minocycline, doxycycline plus albendazole and albendazole alone in their efficacy against onchocerciasis in a randomized, open–label, pilot trial. PLoS Neglected Trop. Dis. 1–22. doi: 10.1371/journal.pntd.0005156 PMC5215804 28056021

[B39] Landmann F. Voronin D. Sullivan W. Taylor M. J. (2011). Anti–filarial activity of antibiotic therapy is due to extensive apoptosis after wolbachia depletion from filarial nematodes. PLoS Pathogens 7 (11), 1–11. doi: 10.1371/journal.ppat.1002351 PMC3207916 22072969

[B40] Leopold S. van Leth F. Tarekegn H. Schultsz C. (2014). Antimicrobial drug resistance among clinically relevant bacterial isolates in sub–Saharan Africa: a systematic review. J. Antimicrob. Chemother. 69 (9), 2337–2353. doi: 10.1093/jac/dku176 24879668

[B41] Li Z. Garner A. L. Gloeckner C. Janda KD C. C. (2011). Targeting the wolbachia cell division protein FtsZ as a new approach for antifilarial therapy. PloS Negl. Trop. Dis. 5, e1411. doi: 10.1371/journal.pntd.0001411 22140592 PMC3226453

[B42] Litt E. Baker M. C. Molyneux D. (2012). Neglected tropical diseases and mental health: A perspective on comorbidity. Trends Parasitol. 28 (5), 195–201. doi: 10.1016/j.pt.2012.03.001 22475459

[B501] Ma C. Yang X. Lewis P. J . (2016). Bacterial Transcription as a Target for Antibacterial Drug Development. Microbiol. Molecular Biol. Revs. 80 (1), 139–160. doi: 10.1128/MMBR.00055-15 PMC4771368 26764017

[B44] Mand S. Debrah A. Y. Klarmann U. Batsa L. Marfo–Debrekyei Y. Kwarteng A. . (2012). Doxycycline improves filarial lymphedema independent of active filarial infection: A randomized controlled trial. Clin. Infect. Dis. 55 (5), 621–630. doi: 10.1093/cid/cis486 22610930 PMC3412691

[B45] Martin R. (1997). Modes of action of anthelmintic drugs. Vet. J. 154 (1), 11–34. doi: 10.1016/S1090-0233(05)80005-X 9265850

[B46] McCarthy J. (2005). Is anthelmintic resistance a threat to the program to eliminate lymphatic filariasis? Am. J. Trop. Med. Hyg. 73, 232–233. doi: 10.4269/ajtmh.2005.73.232 16103580

[B47] McGarry H. F. Egerton G. L. Taylor M. J. (2004). Population dynamics of wolbachia bacterial endosymbionts in brugia malayi. Mol. Biochem. Parasitol. 135 (1), 57–67. doi: 10.1016/j.molbiopara.2004.01.006 15287587

[B48] Mclaren D. J. Worms M. J. (1975). Micro-organisms in filarial larvae (Nematoda). Transactions of the Royal Society of Tropical Medicine and Hygiene, 69(5), 509–514. doi: 10.1016/0035-9203(75)90110-8 1228988

[B49] Micromedex I. (2022). Rifapentine (Oral Route). Pharmacist, American Society of Health, National Library of Medicine.

[B50] Misra N. Sharma M. Raj K. Misra–Bhattacharya S. (2007). Chemical constituents and antifilarial activity of lantana camara against human lymphatic filariid brugia malayi and rodent filariid acanthocheilonema viteae maintained in rodent hosts. Parasitol. 100 (3), 439–448. doi: 10.1007/s00436-006-0312-y 17061115

[B51] Opoku N. Bakajika D. Kanza E. Al E. (2018). Single dose moxidectin versus ivermectin for onchocerca volvulus infection in Ghana, Liberia, and the democratic republic of the Congo: a randomised, controlled, double–blind phase 3 trial. Lancet. 392 (10154), 1207–1216. doi: 10.1016/S0140-6736(17)32844-1 29361335 PMC6172290

[B52] Pfarr K. M. Debrah A. Y. Specht S. Hoerauf A. (2009). Filariasis and lymphoedema. Parasite Immunol. 31 (11), 664–672. doi: 10.1111/j.1365-3024.2009.01133.x 19825106 PMC2784903

[B53] Pichon G. (2002). Limitation and facilitation in the vectors and other as_pects of the dynamics of filarial transmission: the need for vector control against anopheles transmitted filariasis. Ann. Trop. Med. Pa_rasitol 96, S143–S152. doi: 10.1179/000349802125002509 12625927

[B54] Rao R. W. G. (2002). *In vitro* effects of antibiotics on brugia malayi worm survival and reproduction. J. Parasitol. 88, 605–611. doi: 10.1645/0022-3395(2002)088[0605:IVEOAO]2.0.CO;2 12099435

[B55] Richard F. O. J. Amann J. Arana B. Punkosdy G. Klein R. Blanco C. (2007) No depletion of wolbachia from onchocerca volvulus after a shortcourse of rifampin and / or azithromycin no depletion of wolbachia fromonchocerca volvulus after a short course of rifampin and / or azithromycin. Am J Trop Med Hyg 77(5), 872–82. doi: 10.4269/ajtmh.2007.77.878 17984346

[B56] Sabharwal R. Anandharaman M. (2010). Effect of certain antibiotics against filarial parasite brugia malayi *In Vitro* : Possible role of oxidative stress. Indian Journal of Clinical Biochemistry 25 (4), 362–366. doi: 10.1007/s12291-010-0068-0 21966105 PMC2994559

[B57] Schäberle T. F. Schiefer A. Schmitz A. König GM H. A. Pfarr K. . (2014). Corallopyronin A – A promising antibiotic for treatment of filariasis. International Journal of Medical Microbiology 304(1), 72–78. 24079981 10.1016/j.ijmm.2013.08.010

[B58] Schiefer A. Schmitz A. Schäberle T. F. Specht S. Lämmer C. Johnston K. L. . (2012). Corallopyronin a specifically targets and depletes essential obligate wolbachia endobacteria from filarial nematodes *in vivo* . J. Infect. Dis. 206, 249–257. doi: 10.1093/infdis/jis341 22586066 PMC3490692

[B59] Scholar E. (2007). Moxifloxacin. xPharm Compr Pharmacol Ref B978–00805, 1–6. doi: 10.1016/B978-008055232-3.62211-1

[B60] Schwab A. Boakye D. Kyelem D. Prichard R. (2005). Detection of benzi_midazole resistance–associated mutations in the filarial nematode wuchereria bancrofti and evidence for selection by albendazole and ivermectin combination treatment. Am. J. Trop. Med. Hyg. 73, 234–238. doi: 10.4269/ajtmh.2005.73.234 16103581

[B61] Sharma R. Jayoussi G. Tyrer H. E. Gamble J. Hayward L. Guimaraes A. F. . (2016). Minocycline as a re–purposed anti– wolbachia macrofilaricide: superiority compared with doxycycline regimens in a murine infection model of human lymphatic filariasis. Nat. Publ Gr. 6(January), 1–11. doi: 10.1038/srep23458 PMC4800446 26996237

[B62] Simonsen P. E. Pedersen E. M. Rwegoshora R. T. Malecela M. N. Derua Y. A. Magesa S. M . (2010). Lymphatic filariasis control in Tanzania: Effect of repeated mass drug administration with ivermectin and albendazole on infection and transmission. PLoS Neg. Trop. Dis. 4 (6). doi: 10.1371/journal.pntd.0000696 PMC2879369 20532226

[B63] Smith K. Leyden J. J. (2005). Safety of doxycycline and minocycline: A systematic review. Clin. Ther. 27 (9), 1329–1342. doi: 10.1016/j.clinthera.2005.09.005 16291409

[B64] Sodahlon Y. K. Dorkenoo A. M. Morgah K. Nabiliou K. Agbo K. Miller R. . (2013). A success story: Togo is moving toward becoming the first Sub–Saharan African nation to eliminate lymphatic filariasis through mass drug administration and countrywide morbidity alleviation. PloS Negl. Trop. Dis. 7 (4), e2080. doi: 10.1371/journal.pntd.0002080 23593512 PMC3623714

[B65] Specht S. Pfarr K. M. Arriens S. Hu M. P. Klarmann–U. Koschel M. . (2018). Combinations of registered drugs reduce treatment times required to deplete wolbachia in the litomosoides sigmodontis mouse model 1–20. doi: 10.1371/journal.pntd.0006116 PMC5771630 29300732

[B66] Stolk W. A. Walker M. Coffeng L. E. Basáñez M. Vlas S. J. De. (2015). Required duration of mass ivermectin treatment for onchocerciasis elimination in Africa : a comparative modelling analysis. Parasit Vectors, 1–16. doi: 10.1186/s13071-015-1159-9 26489937 PMC4618738

[B67] Taylor M. J. Cross H. F. Bilo K. (2000). Inflammatory responses induced by the filarial nematode brugia malayi are mediated by lipopolysaccharide–like activity from endosymbiotic wolbachia bacteria. J. Exp. Med. 191 (8), 1429–1436. doi: 10.1084/jem.191.8.1429 10770808 PMC2193140

[B68] Taylor M. J. Hoerauf A. Bockarie M. (2010). Lymphatic filariasis and onchocerciasis. Lancet. 376 (9747), 1175–1185. doi: 10.1016/S0140-6736(10)60586-7 20739055

[B69] Thomsen E. K. Sanuku N. Baea M. Satofan S. Maki E L. B. (2016). Efficacy, safety, and pharmacokinetics of coadministered diethylcarbamazine, albendazole, and ivermectin for treatment of bancroftian filariasis. Clin. Infect. Dis. 62 (3), 334–341. doi: 10.1093/cid/civ882 26486704

[B70] Townson S. Hutton D. Siemienska J. Hollick L. Scanlon T. Tagboto S. K. . (2000). Antibiotics and wolbachia in filarial nematodes: antifilarial activity of rifampicin, oxytetracycline and chloramphenicol against onchocerca gutturosa , onchocerca lienalis and brugia pahangi. Ann. Trop. Med. Parasitol. 94 (8), 801–816. doi: 10.1080/00034983.2000.11813605 11214099

[B71] Traore M. O. Sarr M. D. Badji A. Bissan Y. Diawara L. Doumbia K. . (2012). Proof–of–Principle of onchocerciasis elimination with ivermectin treatment in endemic foci in Africa. Final Results of a Study in Mali and Senegal 6(9). doi: 10.1371/journal.pntd.0001825 PMC3441490 23029586

[B72] Turner J. D. Mand S. Debrah A. Y. Muehlfeld J. Pfarr K. Mcgarry H. F. . (2006). Randomized, double–blind clinical trial of a 3–week course of doxycycline plus albendazole and ivermectin for the treatment of wuchereria bancrofti infection. Clin Infect Dis. 42(8), 1081–9. doi: 10.1086/501351 16575724

[B73] Turner J. D. Tendongfor N. Esum M. Johnston K. L. Stuart Langley R. Ford L. . (2010). Macrofilaricidal activity after doxycycline only treatment of onchocerca volvulus in an area of loa loa co–endemicity: A randomized controlled trial. PloS Negl. Trop. Dis. 4 (4), e660. doi: 10.1371/journal.pntd.0000660 20405054 PMC2854122

[B74] Turner H. Walker M. Attah S. Al E. (2015). The potential impact of moxidectin on onchocerciasis elimination in Africa: an economic evaluation based on the phase II clinical trial data. Parasit Vectors 8, 167. doi: 10.1186/s13071-015-0779-4 25889256 PMC4381491

[B75] Upadhya R. Shenoy K. Laxmi Venkateswaran R. (2018). Minocycline: The second important antimicrobial in multidrug−resistant acinetobacter baumanii infections hyperbaric bupivacaine. J. Anaesthesiol Clin. Pharmacol. 34 (3), 46–50. doi: 10.4103/joacp.JOACP 29643650 PMC5885441

[B76] Volkmann L. Fischer K. Taylor M. Hoerauf A. (2003). Antibiotic therapy in murine filariasis (Litomosoides sigmodontis): Comparative effects of doxycycline and rifampicin on wolbachia and filarial viability. Trop. Med. Int. Heal. 8 (5), 392–401. doi: 10.1046/j.1365-3156.2003.01040.x 12753632

[B77] Voronin D. Cook D. A. N. Steven A T. M. (2012). Autophagy regulates wolbachia populations across diverse symbiotic associations. Proc. Natl. Acad. Sci. U S A 109, E1638–E1646. doi: 10.1073/pnas.1203519109 22645363 PMC3382551

[B78] Wanji S. Kengne–ouafo J. A. Esum M. E. Chounna P. W. N. Tendongfor N. Adzemye B. F. . (2015). Situation analysis of parasitological and entomological indices of onchocerciasis transmission in three drainage basins of the rain forest of south West Cameroon after a decade of ivermectin treatment. Parasit Vectors 8(1):1–21. doi: 10.1186/s13071-015-0817-2 25886166 PMC4393872

[B79] WHO (2011). Lymphatic filariasis: report. Geneva, Switzerland: WHO.

[B80] Who (2013). Global programme to eliminate lymphatic filariasis: Managing morbidity and preventing disability (World Heal Organ). Geneva, Switzerland: WHO 1–69. doi: 10.1371/jour

[B81] WHO (2017a). Summary of global update on preventive chemotherapy implementation in 2016: crossing the billion. Wkly Epidemiol. Rec 40, 589–608. Available at: https://apps.who.int/iris/handle/10665/259185. 28984120

[B82] WHO (2017b). Global Vector Control Response 2017–2030. Geneva, Switzerland: WHO.

[B83] Winnen M. Plaisier A. P. Alley E. S. Nagelkerke N. J. D. Oortmarssen G.V. Boatin B. A. . (2002). Can ivermectin mass treatments eliminate onchocerciasis in Africa? Bulletin of the World Health Organization 80 (00), 384–390. 12077614 PMC2567795

[B84] World Health Organization (2004). Global programme for the elimination of lymphatic filariasis. Geneva, Switzerland: WHO.

